# Unlocking the future of optical security with metasurfaces

**DOI:** 10.1038/s41377-021-00589-4

**Published:** 2021-07-14

**Authors:** Jaehyuck Jang, Trevon Badloe, Junsuk Rho

**Affiliations:** 1 Department of Chemical Engineering, Pohang University of Science and Technology (POSTECH), Pohang, 37673 Republic of Korea; 2 Department of Mechanical Engineering, Pohang University of Science and Technology (POSTECH), Pohang, 37673 Republic of Korea; 3 POSCO-POSTECH-RIST Convergence Research Center for Flat Optics and Metaphotonics, Pohang, 37673 Republic of Korea

**Keywords:** Nanophotonics and plasmonics, Metamaterials, Displays, Micro-optics

## Abstract

The complex degrees of freedom of light, such as amplitude, phase, polarization, and orbital angular momentum, make it a prime candidate for use in optical security and encryption. By exploiting the unique characteristics of metasurfaces, exciting new optical security platforms have been demonstrated.

As we enter an age where the protection and security of our personal data are becoming increasingly important, new secure methods of encrypting and sharing information are paramount. Optical security is an excellent candidate with regard to the numerous degrees of freedom of light, such as the phase, amplitude, and polarization. Different combinations of these characteristics of light can be used to produce innovative methods of multiplexing the encryption and decryption of sensitive data. As exciting new methods to control light at will through the use of metamaterials, in particular their 2D form known as metasurfaces, are uncovered, potential applications for increased safety using optical security are becoming a reality.

One such technique is known as computational ghost imaging (CGI)^1^, which is a method of reconstructing encrypted information through indirect measurements using the correlation between the photocurrent of a single-pixel bucket detector and a statistically independent noise pattern. As one of the first demonstrations of combining metasurfaces into this type of optical security, a metahologram was used as the object along with random binary masks^2^. The encoded image can be indirectly transmitted to the receiver through a single measurement of the beam intensity passing through the metasurface and then iteratively discrete random binary masks. Using a sufficiently large sample of measured beam intensity and random binary mask sets, the encrypted image of the metahologram can be decrypted using the CGI algorithm. This first scheme using the properties of light through multiple cascaded 2D maps drew attention to the opportunities for optical security that could be realized through stacks of metasurfaces.

Adding an element of tunability to the metasurface allows for multiple pieces of encrypted information to be passed between parties. In one example, in particular, an orbital angular momentum (OAM)-generating metasurface was cascaded with an OAM-dependent metahologram^3^. The tunable metasurface, made up of interleaved gold and magnesium nanopatterns, produces a vector beam with a different topological charge under H_2_ and O_2_ loading, while the OAM-dependent metasurface reconstructs a hologram based on the topological charge of the incident vector beam. In this way, the two cascaded metasurfaces show holographic images that can be switched with an external stimulus. Furthermore, since each metasurface serves its purpose with its functionality rather than using random masks, the benefits of integrating metasurfaces into optical security applications are highlighted further.

The metasurfaces used to produce metaholograms generally physically recreate the phase masks that have been designed to modulate the incident light in the desired fashion to recreate the encoded image. However, the cascaded phase modulators do not necessarily have to be metasurfaces. Qu et al. demonstrated reprogrammable metasurface holography using the combination of a tunable phase mask through the use of a spatial light modulator (SLM) cascaded with a static metasurface^4^. The key idea is that the phase profile to recreate the desired holographic image is divided into two distinct phase maps that must be arranged correctly to modulate the incident light correctly. Therefore, without prior knowledge about the phase profile encoded into the metasurface, the hologram cannot be correctly reconstructed. The minimum threshold needed for a potential hacker or eavesdropper to steal the information was determined to be higher than that of CGI, increasing the degree of optical security and proving its use for secret sharing. Although the authors insist that this holographic encoding enables reprogrammed metasurfaces, the fixed phase map of the metasurface is static and arbitrary; in other words, it plays no function other than a secondary modulation of the incident beam of light that is needed to recreate the encoded information. Its purpose is solely to enable secret sharing. Similarly, Lu et al. proposed a threshold cryptosystem consisting of three-phase masks^5^. The encoded images can be reconstructed using any two of the three distinct phase masks while acquiring only one provides the eavesdropper with no information. Likewise, this threshold system can play a crucial role in optical security for sharing sensitive information.

Writing in *Science Advances*, Philip Georgi and coauthors demonstrated an optical secret sharing system using cascaded metasurfaces^6^. Each metasurface renders its distinct Fourier hologram, while completely different images are reproduced when they are aligned and cascaded along the propagation direction (Fig. 1a, b). Since metasurfaces are physical devices that can be provided to users, multiple levels of optical security are naturally realized. Anyone trying to intercept sensitive information should acquire not only physical devices but also the correct setup to reconstruct holographic information. These parameters can include the orientation in terms of both translation and rotation, the distance between the cascaded metasurfaces, and the correct operating wavelength. Encoding such details into phase-only metaholograms requires a sophisticated phase retrieval method. The authors, therefore, developed a phase retrieval optimization method based on gradient descent, which effectively produces the phase mask pairs for the individual shares. This optimization technique can be applied to produce any number of combinations of phase masks, which can then be physically realized using metasurfaces, SLMs, or any other type of diffractive optical element. Metasurfaces provide the obvious advantages of high resolution in a compact form factor while also allowing for additional complex multiplexed functionalities that can be used to increase the level of security^7,8^ by designing phase masks that are dependent not only on the translational direction in the *x*–*y* plane but also on the distance between the two shares, as well as various wavelengths of incident light (Fig. 1c). Since the target holograms are arbitrary, it is trivial to produce multiple different subshare sets that reproduce their distinct holographic images alone while combining with a single master share to produce the same hidden optical information, further highlighting the design flexibility of the proposed cascaded metasurface configuration. The amount of information that can be encoded into each set of phase masks is fundamentally limited by the number of pixels available in the master share, underlining the importance of advanced nanofabrication and nanoscale alignment techniques.

**Fig. 1:**
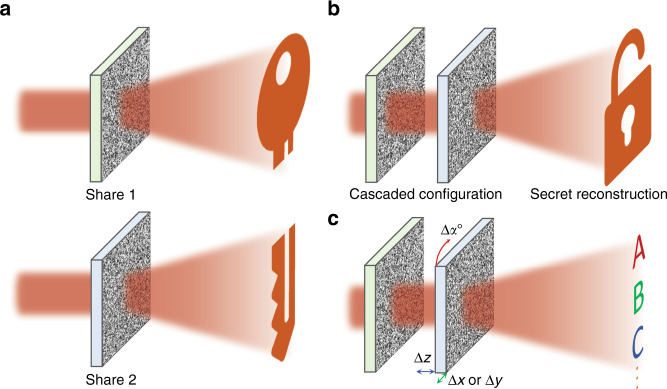
Schematics of an optical secret sharing system using cascaded phase masks. **a** The two computationally designed and encrypted phase masks (Share1 and Share2) reproduce their holographic images. **b** The concealed image is revealed by correctly illuminating the two aligned shares in a cascaded configuration with the correct wavelength of light and gap between them. **c** Incorrect alignment in the translational, longitudinal, and rotational directions does not reproduce the encoded image. However, such spatial sensitivity can be utilized to store more information that can be reconstructed when the shares are in the correct configuration and illuminated with the correct wavelength of light. “Icon made by Pixel perfect from www.flaticon.com”

Cascading two metasurfaces inevitably requires extremely precise alignment since any slight mismatch leads to improper phase accumulation as the light propagates through two phase masks, deteriorating or completely destroying the reconstructed information. However, Georgi et al. suggest an innovative method to utilize this translational sensitivity by introducing the concept of an ‘optical ruler’. By retrieving an extended phase map that covers a wider physical space for one of the metasurfaces, translational multiplexing can be employed to allow the user to measure distances down to the scale of a unit cell of the metasurface, which may allow for translational measurements below the diffraction limit of light.

Among the plethora of metasurface, optical security and encryption applications is one field that is thriving with new ideas and concepts. Zheng et al. recently proposed the idea of computational imaging encryption using a single dual-channel metasurface^9^. The capability of metasurfaces to overcome the intrinsic shortcomings of single-pixel imaging (i.e., CGI) by integrating direct metasurface imaging and indirect computational imaging was proven. Countersurveillance metaoptics were also successfully demonstrated by Xue et al. by taking advantage of irreversible changes in a metal-based metasurface under thermal perturbation^10^. In addition, a photonic security platform using a tunable vectorial holography metasurface to encode and share sensitive information was proposed by Kim et al.^11^. These novel concepts can generate a new era of metasurface-based optical security and encryption.
